# Hypoglycemia: Role of Hypothalamic Glucose-Inhibited (GI) Neurons in Detection and Correction

**DOI:** 10.3389/fphys.2018.00192

**Published:** 2018-03-09

**Authors:** Chunxue Zhou, Suraj B. Teegala, Bilal A. Khan, Christina Gonzalez, Vanessa H. Routh

**Affiliations:** Department of Pharmacology, Physiology and Neurosciences, New Jersey Medical School, Rutgers, The State University of New Jersey, Newark, NJ, United States

**Keywords:** ventromedial hypothalamus, neuronal nitric oxide synthase, perifornical hypothalamus, orexin, oxidative stress, hypoglycemia associated autonomic failure, hypoglycemia unawareness

## Abstract

Hypoglycemia is a profound threat to the brain since glucose is its primary fuel. As a result, glucose sensors are widely located in the central nervous system and periphery. In this perspective we will focus on the role of hypothalamic glucose-inhibited (GI) neurons in sensing and correcting hypoglycemia. In particular, we will discuss GI neurons in the ventromedial hypothalamus (VMH) which express neuronal nitric oxide synthase (nNOS) and in the perifornical hypothalamus (PFH) which express orexin. The ability of VMH nNOS-GI neurons to depolarize in low glucose closely parallels the hormonal response to hypoglycemia which stimulates gluconeogenesis. We have found that nitric oxide (NO) production in low glucose is dependent on oxidative status. In this perspective we will discuss the potential relevance of our work showing that enhancing the glutathione antioxidant system prevents hypoglycemia associated autonomic failure (HAAF) in non-diabetic rats whereas VMH overexpression of the thioredoxin antioxidant system restores hypoglycemia counterregulation in rats with type 1 diabetes.We will also address the potential role of the orexin-GI neurons in the arousal response needed for hypoglycemia awareness which leads to behavioral correction (e.g., food intake, glucose administration). The potential relationship between the hypothalamic sensors and the neurocircuitry in the hindbrain and portal mesenteric vein which is critical for hypoglycemia correction will then be discussed.

## Introduction

Glucose is the primary fuel of the brain. For this reason, neural circuits exist which sense declining glucose levels and restore euglycemia. These circuits evolved to protect against falls in blood glucose during exercise or fasting. In the modern world, they are also the critical defense against life-threatening insulin-induced hypoglycemia (Cryer, 1981). When glucose levels fall below 80 mg/dl a sequential hormonal response is initiated to restore euglycemia. Insulin secretion ceases followed by the stepwise secretion of the gluconeogenic hormones glucagon, epinephrine, cortisone and growth hormone as glucose levels continue to fall toward ~60 mg/dl (Amiel et al., 1988; Mitrakou et al., 1991). This hormonal response is referred to as the counterregulatory response to hypoglycemia (CRR). Decreasing blood glucose levels below 60 mg/dl also initiates neurogenic (e.g., palpitations, sweating) followed by neuroglycopenic (tired/drowsy, difficulty thinking) symptoms near 50 mg/dl which together comprise hypoglycemia awareness (Mitrakou et al., 1991). Hypoglycemia awareness promotes behavioral responses such as feeding or glucose administration (Cryer, 1981; Amiel et al., 1988).

Recurrent iatrogenic hypoglycemia (RH) is the chief barrier to maintaining optimal glycemic control using intensive insulin therapy in type 1 and advanced type 2 diabetes mellitus. RH leads to the deleterious syndromes known as hypoglycemia associated autonomic failure (HAAF) and hypoglycemia unawareness. During HAAF, the glycemic threshold for detection of hypoglycemia shifts to lower glucose levels. When this occurs CRR initiation can be dangerously delayed and blunted in magnitude. Similarly during hypoglycemia unawareness neurogenic and neuroglycopenic symptoms do not warn an individual of impending hypoglycemia and thus prevent behavioral correction (Amiel et al., 1988; Mitrakou et al., 1991; Cryer, 2013). Hypoglycemia unawareness was thought to result from failure of autonomic activation and potentially autonomic neuropathy (Towler et al., 1993). However, more recent studies suggest that heightened arousal is necessary to recognize and interpret the peripheral neurogenic symptoms. This led to the hypothesis that incorrect interpretation of peripheral symptoms of hypoglycemia is a key component of hypoglycemia unawareness (Otlivanchik et al., 2015, 2016).

Robust initiation of corrective mechanisms is particularly important for diabetic patients since even without clinical HAAF glucagon release in response to hypoglycemia is nearly absent in type 1 and advanced type 2 diabetic patients (Bottini et al., 1997; Segel et al., 2002; Cryer, 2005; Israelian et al., 2006; Siafarikas et al., 2012; Oyer, 2013). Thus, improving and preserving proper hypoglycemia detection is critical for diabetes management. Animal studies have described glucose sensors throughout the brain and in many peripheral tissues (Routh et al., 2012). However, those within the hypothalamus have been the most extensively studied, especially in relation to HAAF and hypoglycemia unawareness (Routh, 2003; Song and Routh, 2006; Kang et al., 2008; Fioramonti et al., 2010; Chan et al., 2013; Fan et al., 2015; Otlivanchik et al., 2015, 2016). In this article we will focus on hypothalamic sensors and their involvement in these pathologies resulting from RH. We will then explore their potential interaction with the important neural circuits of the hindbrain and portal vein of the liver which are also key for the CRR (Ritter et al., 2011; Donovan and Watts, 2014).

## Hypothalamic glucose sensing and hypoglycemia detection

Strong evidence in rodents supports a role for ventromedial hypothalamus (VMH) glucose sensors in the hormonal CRR (Borg et al., 1994, 1995, 1997; Tong et al., 2007) as well as in glucoprivic feeding (Dunn-Meynell et al., 2009). The VMH possesses two major subtypes of glucose-sensing neurons (Anand et al., 1964; Oomura et al., 1964). Glucose-excited (GE) neurons increase while glucose-inhibited (GI) neurons decrease their activity as glucose levels rise (Ashford et al., 1990; Song et al., 2001). As mentioned above the ability to sense glucose has been observed throughout the brain in neurons as well as in glial cells (Levin, 2000; Marty et al., 2005; Zhou et al., 2010; Frayling et al., 2011; Melnick et al., 2011; Routh et al., 2012). It is true that any glucose sensing cell within the CNS may potentially contribute to the regulation of the CRR. However, the glucose sensitivity of VMH glucose sensing neurons, especially that of GI neurons, parallels the ability of the brain to sense and respond to hypoglycemia (McCrimmon et al., 2006; Song and Routh, 2006; Kang et al., 2008; Diggs-Andrews et al., 2010; Fioramonti et al., 2010, 2013). This has important implications for preserving the CRR during HAAF and diabetes. Thus, this perspective focuses primarily on these neurons. However, this does not preclude important roles for other central glucose sensors in regulating the CRR.

### VMH glucose sensing neurons

Like the pancreatic β-cell, glucose sensing by VMH GE neurons depends on a low-affinity hexokinase IV isoform, glucokinase (GK) and the ATP-sensitive K^+^ channel (K_ATP_)(Ashford et al., 1990; Dunn-Meynell et al., 2002; Kang et al., 2006). GK also mediates glucose sensing in VMH GI neurons (Dunn-Meynell et al., 2002; Kang et al., 2006). However, in GI neurons, decreased glucose activates the cellular fuel sensor AMP activated protein kinase (AMPK). AMPK phosphorylates the neuronal nitric oxide (NO) synthase (nNOS) leading to NO production. NO then binds to its receptor, soluble guanylyl cyclase (sGC), which increases cyclic guanosine monophosphate (cGMP) production. Increased cGMP causes a further activation of AMPK. This cGMP mediated activation of AMPK leads to closure of a chloride channel, possibly the cystic fibrosis transmembrane regulator (CFTR), depolarization, and increased neuronal activity (Murphy et al., 2009a). These studies demonstrate that NO production is required for depolarization of GI neurons in low glucose. However, NO is a diffusible gas which acts as a retrograde signal at synapses and plays a role in plasticity (Edelmann et al., 2007; Dejanovic and Schwarz, 2014). NO diffusion also regulates neuroendocrine processes via recruitment of adjacent neurons (Bellefontaine et al., 2014). Whether NO diffusion within synapses or to adjacent cells plays a role in glucose sensing is not known.

**Figure 1:**
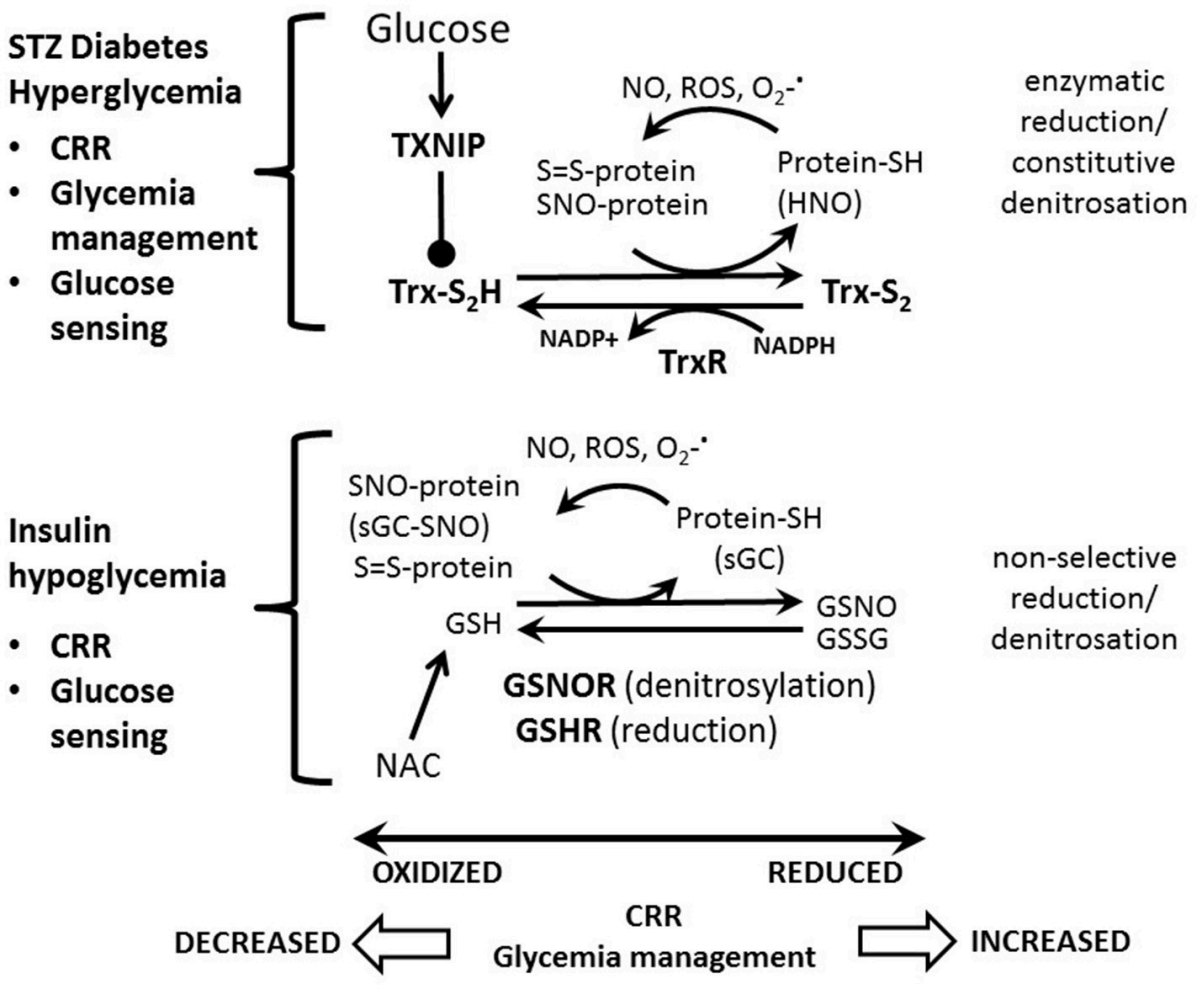
Model for the respective roles of the VMH glutathione and thioredoxin systems in HAAF and diabetes. Increased nitric oxide (NO), reactive oxygen species (ROS) and/or superoxide (${\text{O}}_{2}^{-}\cdot$) cause protein S-nitrosation (SNO-protein) and oxidation (S = S-protein). We have shown that soluble guanylyl cyclase is S-nitrosated (sGC-SNO) during hypoglycemia leading to impaired glucose sensing and counterregulatory response (CRR). Reduced glutathione (GSH) causes a non-selective reduction and/or denitrosation of oxidized proteins (protein-SH; sGC) becoming oxidized (GSSG) or nitrosated (GSNO) itself in the process. GSH is regenerated by the enzymes glutathione reductase (GSHR) and nitrosated glutathione reductase (GSNOR) (Nordberg and Arnér, 2001). Oral administration of the glutathione precursor, N-acetylcysteine (NAC), completely prevents hypoglycemia associated autonomic failure (HAAF) and preserves glucose sensing by VMH GI neurons in non-diabetic but not diabetic animals. Reduced thioredoxin (Trx-S_2_H) enzymatically catalyzes protein reduction and denitrosation becoming oxidized in the process (Trx-S_2_). During denitrosation HNO is released. Trx-S_2_H is regenerated by the enzyme thioredoxin reductase (TrxR) in an NADPH dependent fashion. The glucose activated enzyme, thioredoxin interacting protein (TXNIP) reduces Trx-S_2_H activity (Stoyanovsky et al., 2005). VMH thioredoxin overexpression completely normalizes glucose sensing by GI neurons and the CRR in diabetes alone but not when HAAF occurs during diabetes. VMH thioredoxin overexpression also reduces the insulin required to manage hyperglycemia in diabetes. Enzyme abbreviations are bold-faced.

The ability of VMH GI neurons to be activated by low glucose closely parallels the magnitude of the CRR. For example, as seen for CRR initiation, glucose levels must fall further after RH before GI neurons are activated (Song and Routh, 2006). AMPK, nNOS and sGC inhibition in the perfusion solution *in vitro* and in the VMH *in vivo*, respectively, blocks activation of GI neurons in low glucose and impairs the CRR (McCrimmon et al., 2008; Murphy et al., 2009a; Fioramonti et al., 2010). Hypo- or hyperglycemia induced-oxidative/nitrosative stress may underlie both the blunted response of VMH GI neurons to decreased glucose and CRR impairment (Colombani et al., 2009; Fioramonti et al., 2013). Oxidative and nitrosative stress cause s-nitrosation of sGC (and other proteins)(Jaffrey et al., 2001). Nitrosated sGC is resistant to NO (Sayed et al., 2007). Insulin-induced hypoglycemia increases sGC s-nitrosation and VMH injection with a nitrosating agent impairs the CRR. Furthermore, reducing oxidative stress with the glutathione precursor, N-acetylcysteine, in non-diabetic rats completely prevented altered glucose sensing by VMH GI neurons and CRR impairment during HAAF (Fioramonti et al., 2013). Diabetic hyperglycemia also induces oxidative stress and reduces both activation of VMH GI neurons by decreased glucose and glucagon secretion during hypoglycemia (Cryer et al., 2003; Canabal et al., 2007a; Cardoso et al., 2010; Zhou and Routh, 2017). However, N-acetylcysteine did not normalize glucose sensing and CRR initiation in diabetic animals (Zhou and Routh, 2017). Diabetic hypoglycemia is associated with impairment of the VMH thioredoxin antioxidant system via activation of the inhibitory enzyme, thioredoxin interacting protein or TXNIP (Blouet and Schwartz, 2011). VMH thioredoxin overexpression completely normalized GI neuronal glucose sensing, glycemia recovery, and glucagon secretion in rats with type 1 diabetes mellitus. However, VMH thioredoxin overexpression did not prevent HAAF in these animals (Zhou and Routh, 2017). These findings are interesting for two reasons. First, impaired glucagon secretion in diabetes is often thought to be due to local factors at the level of the alpha cell. However, these studies suggest a direct effect of the VMH on pancreatic alpha cell function during the CRR. Second, the effects of N-acetylcysteine were more pronounced during RH whereas VMH thioredoxin overexpression was more effective in diabetes. Neither antioxidant system alone compensated for HAAF when it occurred during diabetes. This suggests that a combination of antioxidants might be necessary to preserve the CRR when HAAF occurs during diabetes. Figure 1 illustrated the respective roles of the VMH glutathione and thioredoxin systems in HAAF and diabetes.

VMH thioredoxin overexpression had 2 additional interesting and unexpected effects. First, while virtually all animals injected with the control vector developed diabetes after one injection of the β-cell toxin streptozotocin, one quarter of the VMH thioredoxin overexpressing animals did not (Zhou and Routh, 2017). This suggests that the VMH has a protective role on the pancreatic β-cell, perhaps by reducing inflammation. However, it should be noted that a second injection of toxin led to diabetes in the resistant animals. Nevertheless, this putative protective effect of enhanced VMH antioxidant defense on the pancreas deserves further study. The second unexpected effect of VMH thioredoxin overexpression was a reduction in the amount of insulin required to manage blood glucose levels (Zhou and Routh, 2017). Whether this is due to reduced basal hepatic glucose output, increased glucose uptake by insulin sensitive tissues, or other changes in metabolism is not known. However, this finding is consistent with a role of the VMH in peripheral insulin sensitivity and glucose homeostasis (Steffens et al., 1988; Martins et al., 2016; Coutinho et al., 2017). Moreover a role for VMH oxidative status in glucose homeostasis is consistent with a recent report that uncoupling protein regulates VMH glucose sensing and peripheral glycemia by decreasing oxidative stress (Toda et al., 2016).

Further support for a role of VMH GI-nNOS neurons in the CRR derives from their relationship with glutamate signaling. The VMH possesses high expression of mRNA encoding the vesicular glutamate transporter VGLUT2, a marker for glutamate neurons (Ziegler et al., 2002). Virtually all VMH nNOS neurons are glutamatergic (Chachlaki et al., 2017). Since we find that 95% of VMH GI neurons produce NO in decreased glucose and GI neurons are absent in mice lacking nNOS, it is very likely that VMH GI neurons are glutamatergic (Canabal et al., 2007b; Fioramonti et al., 2010). Mice lacking VGLUT2 selectively in steroidogenic factor 1 neurons that mark the VMH have defective CRR to insulin-induced hypoglycemia (Tong et al., 2007). Taken together, these data lead us to hypothesize that maintaining normal oxidative balance in VMH glutamatergic nNOS expressing GI neurons is necessary for the CRR and potentially other aspects of glucose homeostasis.

### Orexin glucose sensing neurons and hypoglycemia unawareness

Hypothalamic orexin neurons are also GI neurons (Burdakov et al., 2005). Perifornical hypothalamus (PFH) orexin neurons play a role in epinephrine secretion during hypoglycemia (Otlivanchik et al., 2015; Korim et al., 2016). Orexin neurons may also contribute to hypoglycemia awareness. Otlivanchik et al showed that one episode of insulin-hypoglycemia in the conditioned side of a conditioned place preference (CPP) box reversed the preference for that side of the box on the subsequent day. They interpreted this finding to mean that the animal was aware of hypoglycemia, associated it with the formerly preferred side of the box and developed an aversion to that side. Interestingly, 3 consecutive daily episodes of hypoglycemia *in the home cage* prevented subsequent hypoglycemia on the preferred side from disrupting the CPP. This suggests that the animal exhibited hypoglycemia unawareness and thus did not develop an aversion. Interestingly, systemic injection of a brain permeant orexin antagonist mimicked hypoglycemia unawareness (Otlivanchik et al., 2016). PFH orexin neurons facilitate arousal via their projections to the tuberomammillary nucleus histamine neurons (Sundvik and Panula, 2015). Thus, PFH orexin-GI neurons may play a role in hypoglycemia awareness and be a target for treating hypoglycemia unawareness.

An interesting characteristic of the glucose sensitivity of VMH and orexin GI neurons is regulation by metabolic state. For example, fasting increases, while the satiety hormone leptin decreases the activation of VMH and orexin GI neurons, as well as that of arcuate nucleus neuropeptide Y expressing GI neurons, by decreased glucose (Murphy et al., 2009b; Sheng et al., 2014). The hunger hormone ghrelin increases the activation of orexin-GI neurons in low glucose (Sheng et al., 2014). Thus, during energy deficit when low glucose is a greater threat hypoglycemia may produce a stronger activation of hypothalamic GI neurons. This would enable a more robust response to hypoglycemia despite diminished energy reserves.

## Relationship between hypothalamic, hindbrain and portal-mesenteric vein (PMV) glucose sensors

Glucose sensors in the hindbrain and PMV are critical for the CRR as detailed in several comprehensive review articles (Ritter et al., 2011; Routh et al., 2012; Donovan and Watts, 2014). Ritter and colleagues show that specific clusters of catecholamine neurons within the C1 cell groups (C1r, C1m, A1/C1) of the rostral ventral lateral medulla (RVLM) in rodents are essential for individual components of hypoglycemia correction including epinephrine and corticosterone secretion as well as glucoprivic feeding (Ritter et al., 1981, 1998, 2001, 2006; Li et al., 2017). The feeding and corticosterone response is mediated by forebrain projections to hypothalamus (i.e. paraventricular nucleus, PFH) whereas the adrenomedullary response is mediated by bulbospinal projections (Ritter et al., 2001, 2006; Li et al., 2015b). Recent work by this group has shown that RVLM catecholamine neurons reciprocally innervate PFH orexin neurons in order to control glucoprivic feeding responses (Li et al., 2015a,b). These authors present the intriguing hypothesis that this interaction may enable the orexin system to coordinate arousal with feeding behavior.

Glucose sensors within the PMV are also essential for the hormonal CRR in experimental models (Donovan et al., 1994; Hevener et al., 2000; Fujita et al., 2007; Donovan and Watts, 2014). Interestingly, the role of the PMV glucose sensor in the CRR is dependent on the rate of glucose decline. That is, PMV glucose sensors dominate during slow-onset hypoglycemia “(≤1 mg/dL • min^−1^).” In contrast, CNS sensors dominate when glucose levels fall quickly (≥2 mg · dl^−1^·min^−1^) (Matveyenko et al., 2007; Saberi et al., 2008; Bohland et al., 2014). The former corresponds to a drop in blood glucose from euglycemia (~100 mg/dl) to hypoglycemia (60 mg/dl) within approximately 60 min. while in the latter blood glucose would decrease to 60 mg/dl within 20 min. While slower rates of decline predominate during insulin therapy in humans, rapid decline occurs at an incidence of ~30% (Kovatchev et al., 2005). An additional issue to be considered when interpreting these data is that the studies of the relative role of PMV and CNS glucose sensors on the CRR were done in non-diabetic controls. Thus, whether the starting glycemia plays a role in CNS vs. peripheral detection is not known. Interestingly, hyperglycemia *per se* significantly reduces activation of VMH GI neurons in low glucose (Canabal et al., 2007a).

The cellular mechanism of PMV glucose sensing is still unknown. However, the effect of PMV glucose sensors on the sympathoadrenal response during slow-onset hypoglycemia is dependent on sympathetic afferents to the hindbrain (Bohland et al., 2014). Moreover, hindbrain catecholamine projections to the hypothalamus are essential for sympathoadrenal activation and glucagon secretion during slow- but not fast-onset hypoglycemia (Jokiaho et al., 2014). Together, these data suggest that an interaction between glucose sensors in the PMV and the hypothalamically projecting hindbrain catecholamine neurons is required for the secretion of epinephrine and glucagon when glucose levels fall slowly. In contrast, the hypothalamic glucose sensors dominate when glucose levels fall quickly. It is also possible that any or all of these sensors play a role in the inhibition of insulin secretion during hypoglycemia.

## Perspective

Clearly, hypoglycemia is a severe threat to the brain and therefore multiple neurocircuits have evolved to sense and restore euglycemia. The data discussed in this review suggest hierarchical control of the CRR and feeding responses to hypoglycemia. A model for such a hierarchical organization can be found in an excellent recent review (Donovan and Watts, 2014). These data also suggest some degree of specificity in control of different aspects of the response to hypoglycemia by the different glucose sensing regions. That is, slow-onset hypoglycemia is primarily sensed by the PMV and information is sequentially relayed to the hindbrain catecholamine neurons and hypothalamus in order to increase glucagon and epinephrine secretion. It is likely that this information undergoes integration and further processing at the level of the hindbrain, given the response of the catecholamine neurons to glucoprivic agents. In addition activation of these hindbrain glucose sensors initiates feeding and corticosterone responses through hypothalamic projections. On the other hand when glucose levels fall rapidly, the threat to the brain is more severe and hypothalamic glucose sensors are fully capable of restoring euglycemia.

Based on our data we hypothesize that even under slow-onset hypoglycemia the VMH glucose sensors integrate the information and potentially enhance the CRR during energy deficit. That is, VMH nNOS (glutamatergic?) GI neurons may be important in setting the gain of the response to compensate for the current energetic status of the body. In particular, the degree of activation of VMH nNOS GI neurons in low glucose is closely coupled to the magnitude of the hormonal CRR. These GI neurons are particularly sensitive to oxidative stress and enhancing antioxidant defenses during either RH or diabetes is sufficient to preserve the CRR. Further work needs to be done in order to determine whether this strategy will be effective when HAAF occurs during diabetes. Interestingly, enhancing VMH antioxidant defenses appears to be very important for restoring glucagon responses during early diabetes as well as aiding in glycemic management. In the future it would be interesting to determine whether GI neurons are playing a role in both of these VMH functions. It will also be important to determine whether the effects of thioredoxin persist in type 2 diabetes mellitus or in other models of type 1 diabetes. Finally, the PFH orexin-GI neurons may play a role in hypoglycemia awareness by coupling arousal and behavioral responses during hypoglycemia.

## Author contributions

CZ, ST, BK, and CG wrote portions of the first draft with CZ making the greatest contribution. VR revised the first draft critically for important intellectual content, wrote the Perspectives section and generated the final draft of the manuscript.

### Conflict of interest statement

The authors declare that the research was conducted in the absence of any commercial or financial relationships that could be construed as a potential conflict of interest.
